# Eating habits and lifestyle behaviors during COVID-19 lockdown: The Lebanese experience

**DOI:** 10.1371/journal.pone.0284526

**Published:** 2023-04-19

**Authors:** Suzan Haidar, Michelle Cherfan, Souheil Hallit, Mohamad Rahal, Jihan Safwan

**Affiliations:** 1 Faculty of Arts and Sciences, Department of Nutrition and Food Sciences, Lebanese International University, Beirut, Lebanon; 2 Population Health Division, Gilbert and Rose-Marie Chagoury School of Medicine, Lebanese American University, Byblos, Lebanon; 3 INSPECT-LB (Institut National de Santé Publique, d’Épidémiologie Clinique et de Toxicologie-Liban), Beirut, Lebanon; 4 School of Medicine and Medical Sciences, Holy Spirit University of Kaslik, Jounieh, Lebanon; 5 Applied Science Research Center, Applied Science Private University, Amman, Jordan; 6 Research Department, Psychiatric Hospital of the Cross, Jal Eddib, Lebanon; 7 School of Pharmacy, Lebanese International University, Beirut, Lebanon; University of Petra (UOP), JORDAN

## Abstract

**Objectives:**

This study aimed to assess dietary intake and lifestyle habits followed during the COVID-19 pandemic and subsequent lockdowns, as well as the level of adherence to the Mediterranean diet (MD), among a sample of the Lebanese population.

**Methods:**

A cross-sectional study was conducted during the government-enforced lockdown. A validated, online, questionnaire was used to collect information about dietary and lifestyle habits. The Mediterranean diet adherence screener (MEDAS) was used to assess adherence to the MD.

**Results:**

A total of 1684 participants responded to the survey. Their mean age was 23.92 ± 7.62 years, and 70.4% were females. Approximately a third of the participants reported that their dietary habits did not change and 42.3% acknowledged that their eating habits deteriorated during the lockdown. Participants smoked less and slept more during the lockdown compared to before it. Approximately 19.2% of the sample reported low adherence to the MD whereas 63.9% and 16.9% described moderate and high adherence respectively. Only age was significantly associated with higher MD adherence.

**Conclusion:**

Dietary intake and MD adherence were suboptimal during the COVID-19 lockdown among the sample of the Lebanese population. It is critical that the Lebanese government implements public health programs in order to promote awareness about the importance of adhering to a healthy lifestyle and making appropriate dietary and lifestyle choices.

## Background

In 2019, the SARS-CoV-2 virus emerged and quickly reached pandemic proportions. It manifested as a severe acute respiratory syndrome and the condition became known as COVID-19 [1]. Because of this highly contagious virus, countries all over the world resorted to lockdown and quarantine practices with the hope of controlling infection rates and preventing healthcare systems from collapsing.

Consequently, people had to stay home, work remotely when possible, and students were educated through virtual platforms. However, such preventive measures were not without consequences. It is well known that lockdowns, enforced through physical distancing and self-isolation, have an impact on dietary intake and lifestyle habits, which have an impact on health such as smoking, alcohol consumption and sleep. Such effects are not always positive [2,3]. Additionally, social distancing and isolation have a negative effect on mental health, which can be manifested through increased anxiety, depression, poor sleep quality and decreased physical activity [4].

Accordingly, alterations in macronutrient intake can result. It has been observed that stressed and bored individuals [5] may have an augmented preference for energy-dense foods, particularly those high in sugar and fat [6] since the intake of simple carbohydrates can have a positive effect on mood as it increases serotonin production [7]. Furthermore, lockdowns often result in decreased physical activity [8], which further increases stress levels and exacerbates health problems [9].

In Lebanon, the government adopted strict lockdown practices, with the aim of controlling COVID-19 infection numbers and preventing the virus from spreading. The country was simultaneously going through a severe economic crisis which impacted citizens at every level [10]. Even before such circumstances, the Lebanese people had already shifted towards consuming an atherogenic diet coupled with the alarming increase in nutrition-related cardiovascular risk factors [11].

Thus, it is of no doubt that following a healthy dietary pattern, during such turbulent times, is necessary to ward off disease and decrease inflammation and oxidative stress [12]. There is an abundance of evidence that proves that following the Mediterranean diet (MD) can be key in fighting immune-mediated inflammatory responses [13]. The MD has proven to be one of the healthiest and most sustainable diets for both the prevention and treatment of a myriad of diseases, notably cardiovascular disease and inflammation. It is a diet that is low in saturated fat and simple sugars and simultaneously high in plant protein, whole grains, and monounsaturated fats [14].

Numerous studies have assessed adherence to MD before and during the pandemic [15–18]. The Mediterranean Diet Adherence Screener (MEDAS) is one of the most commonly used tools to assess MD adherence [19–22]. It was originally created for use in the PREDIMED study and has proven to be a valid and reliable tool to address MD adherence among different populations. The screener consists of 14 questions, 12 of which inquire about food consumption and the other two about food habits as related to MD [23]. Each of the 14 items is scored with either 1 point to indicate adherence or 0 point to indicate non-adherence. Higher scores indicate higher adherence [24]. During the lockdown, a study conducted in Cyprus proved moderate adherence to MD [16], whereas, the highest scores during the pandemic lockdown were found among the Spanish population [17]. Additionally, an Italian study revealed a moderate adherence to the MD (scores ranging between 6 and 9) in the majority of respondents [3]. Similarly, Czarniecka-Skubina and colleagues estimated that among a sample of Polish adults, half changed their dietary habits significantly secondary to the pandemic [25]. These score differences may be due to a variety of geographical factors as well as sociocultural characteristics of each population influencing the implementation or adherence to the MD in each country [26]. To our knowledge, no previous study assessed MD adherence among the Lebanese population, especially during an atypical period such as domiciliary confinement worsened by an economic burden in the country.

Therefore, our study aimed to identify the dietary intakes among a Lebanese sample as related to the MD diet during the COVID-19 pandemic and subsequent lockdowns. It also intended to assess other health habits such as smoking and sleep. Such information would be vital to stakeholders, especially public health professionals, so they can create evidence-based public health programs, specifically directed towards the Lebanese population should similar circumstances arise in the future.

## Methods

### Ethics approval and consent to participate

The research was carried out in strict accordance with national and international standards, as well as the Declaration of Helsinki (2000). Before participating in the study, all participants were fully informed about the study’s objectives and were required to approve the consent form. Agreeing with the statement and completion of the entire questionnaire was regarded as informed consent to participate. Participants filled out a survey that was directly linked to the Google platform. To protect and preserve confidentiality, participants’ personal information was anonymized, which prevents sensitive personal data from being traced in any manner. The study was approved by the Research and Ethics Committee of the School of Pharmacy at the Lebanese International University.

### Design, setting, and participants

A cross-sectional study was conducted between the months of January and February 2021, when the Lebanese government started enforcing a nationwide lockdown and round-the-clock curfew, in an attempt to curb the spread of coronavirus infections that got out of control following the holiday season. An anonymous online, validated questionnaire was used to collect information about people’s eating and lifestyle habits during the lockdown in Lebanon’s eight governorates (Mohafazat). The inclusion criteria were that participants were at least 16 years old whereas the study excluded those who were below 16 years of age.

### Minimal sample size calculation

According to the G-power software (R^2^ deviation from zero), based on an effect size f^2^ = 0.02, a 95% confidence level, a 95% power, and a total of 10 factors to be entered in the multivariable analysis, a minimum sample size of 1229 people was required to provide adequate statistical power.

### Data collection

Google forms was used to create an online survey, which was conducted in Arabic. The link to the questionnaire was forwarded to the investigators’ personal connections and was spread through institutional and private social networks. Unlike probabilistic sampling, this form of administration produces a statistical collective whose population parameters cannot be controlled. It was, however, entirely beneficial for the research objectives since it allowed for the widespread distribution of the survey questionnaire during a time when there were significant territorial limits due to the pandemic.

### Questionnaire

The questionnaire was created using Google Forms (https://docs.google.com/forms/d/16Gw17zFbRklQDQi7na6FDv7QmlmSt6Uh1Z2v0gUDNz4/prefill) and was adapted from a study conducted by Di Renzo on eating habits and lifestyle changes in Italy [15] (questionnaire publicly available in the before-mentioned reference). The questionnaire was divided into three parts and included 46 questions divided as follows:

- Part A: Demographic information including gender, age, area of residence, place of living, reported weight and height
- Part B: Information on dietary habits:
  1. adherence to the MD, as measured by the validated 14-item Mediterranean diet adherence screener (MEDAS), with a score range of 0 to 14 points and where higher scores indicate more adherence [20]
  2. daily or weekly consumption of specific foods by asking how much the person eats a specific food per day/week during the COVID-19 pandemic (for example: *How many portions of milk or yogurt do you consume per day during the COVID-19 lockdown period*? *(1 serving = 150 ml in a cup or 125 g a jar)*), frequency of food consumption by asking the person if he eats more or less of specific food during the lockdown (for example: *During the COVID-19 lockdown period*, *which of these foods are you consuming MORE than before*?*)*, as well as, the number of meals consumed per day (for example: *Did you change the number of daily meals*, *during the COVID-19 lockdown period*?*)*
- Part C: Lifestyle habits including grocery shopping, smoking, sleeping hours, and physical activity

The MEDAS questionnaire was used to determine the level of adherence to the MD. Participants were divided into three groups based on their MEDAS scores: (i) low adherence (score 0–5), (ii) medium adherence (score 6–9), and (iii) high adherence (score ≥10) to the MD, with differences in compliance rates determined for each food.

### Statistical analysis

Statistical analysis was performed using SPSS v.22 (IBM, Chicago, IL, USA). Cronbach’s alpha value was calculated for the MEDAS scale. There were no missing data since all questions were required in the online form. The normality of distribution of the MEDAS score was confirmed via a calculation of the skewness and kurtosis; values for asymmetry and kurtosis between -1 and +1 are considered acceptable in order to prove normal univariate distribution [27]. The marginal homogeneity test was used to examine the relationship between categorical variables, whereas the Student t-test and ANOVA were done to look for differences between two and three or more means respectively. Pearson’s test was used to correlate two continuous variables. Finally, a linear regression was conducted taking the MEDAS score as the dependent variable; independent variables entered in the final model were those that showed a p<0.25 in the bivariate analysis [28]. P<0.05 was considered statistically significant.

## Results

A total of 1684 participants enrolled in the study. Their mean age was 23.92 ± 7.62 years, with 70.4% being females. More details about the sociodemographic characteristics are reported in Table 1. The Cronbach’s alpha value for the MEDAS scale in this study was 0.43.

**Table 1:** Sociodemographic characteristics of the participants (N = 1684).

Variable	n (%)
**Gender**	
Male	499 (29.6%)
Female	1185 (70.4%)
**Governorate (Mouhafaza)**	
Beirut	429 (25.5%)
Mount Lebanon	289 (17.2%)
North	251 (14.9%)
South	372 (22.1%)
Bekaa	343 (20.4%)
**Marital status**	
Single/ divorced/ widowed	1449 (86.0%)
Married	235 (14.0%)
**Body Mass Index categories**	
Underweight	105 (6.3%)
Normal	1049 (63.4%)
Overweight	365 (22.1%)
Obese	136 (8.2%)
	**Mean ± SD**
Age (in years)	23.92 ± 7.62
Body Mass Index (kg/m^2^)	23.57 ± 4.61

The mean MEDAS score was 7.42 ± 2.26 (media = 7); 323 participants (19.2%) had low adherence to the MD, whereas 1076 (63.9%) and 285 (16.9%) respondents had moderate and high adherence respectively. Details about the number of portions consumed per day of certain food items during the lockdown are summarized in Table 2.

**Table 2:** Description of the MEDAS items and the number of portions per day of certain food items consumed during the lockdown.

Variable	n (%)
**Pasta and cereals (1 medium portion = 80 g per day)**	
None	121 (7.2%)
Half portion	310 (18.4%)
1 portion	769 (45.7%)
2 portions	359 (21.3%)
3 or more portions	125 (7.4%)
**Bread (1 medium portion = 80 g or 2 slices per day)**	
None	136 (8.1%)
Half portion	344 (20.4%)
1 portion	572 (34.0%)
2 portions	435 (25.8%)
3 or more portions	197 (11.7%)
**Milk or yogurt (1 serving = 150 ml in a cup or 125 g a jar per day)**	
None	407 (24.2%)
Half portion	523 (31.1%)
1 portion	523 (31.1%)
2 portions	174 (10.3%)
3 or more portions	57 (3.4%)
**Cheese or dairy products (1 portion of dairy product = 100 g; 1 portion of matured cheese = 50 g per week)**	
None	134 (8.0%)
Half portion	405 (24.0%)
1 portion	537 (31.9%)
2 portions	311 (18.5%)
3 or more portions	297 (17.6%)
**Number of eggs per week**	
0	273 (16.2%)
1	244 (14.5%)
2	514 (30.5%)
4	333 (19.8%)
5 or more	320 (19.0%)
**MEDAS items**	
Item 1 (yes)	877 (52.1%)
Item 2 (yes)	779 (46.3%)
Item 3 (yes)	1230 (73.0%)
Item 4 (yes)	980 (58.2%)
Item 5 (yes)	913 (54.2%)
Item 6 (yes)	783 (46.5%)
Item 7 (yes)	917 (54.5%)
Item 8 (yes)	199 (11.8%)
Item 9 (yes)	1015 (60.3%)
Item 10 (yes)	408 (24.2%)
Item 11 (yes)	1013 (60.2%)
Item 12 (yes)	923 (54.8%)
Item 13 (yes)	1070 (63.5%)
Item 14 (yes)	1388 (82.4%)

The results revealed that 556 participants (33.0%) reported no change in their dietary habits, 713 respondents (42.3%) admitted that their eating habits became less healthy, whereas 415 (24.6%) believed that their eating habits improved. Description of the food items that were consumed more often during the lockdown is summarized in Table 3. Participants reported increased consumption of fish during the lockdown most (38.4%), followed by sweets, fruits, nuts and meat products respectively.

**Table 3:** Description of the food items that were consumed more during the lockdown compared to before it.

Variable	n (%)
Fish (yes)	646 (38.4%)
Sweets (yes)	363 (21.6%)
Fruits (yes)	299 (17.8%)
Nuts (yes)	286 (17.0%)
Meat products (yes)	284 (16.9%)
Alcoholic drinks (yes)	246 (14.6%)
Fresh vegetables (yes)	226 (13.4%)
Bread (yes)	208 (12.4%)
Cow milk and yogurt (yes)	202 (12.0%)
Eggs (yes)	174 (10.3%)
Dairy products (yes)	162 (9.6%)
Coffee (yes)	112 (6.7%)

A significantly higher percentage of participants reported smoking less than five cigarettes per day during the lockdown as compared to the period before the pandemic, whereas a significantly higher percentage of participants acknowledged sleeping for more than 9 hours per night during the lockdown (Table 4).

**Table 4:** Changes in smoking and sleep habits during the lockdown compared to before it.

Variable	Before pandemic	During lockdown	*p*
**Smoking**			**<0.001** *
No	1181 (70.1%)	1190 (70.7%)	
Yes, <5 cigarettes per day	253 (15.0%)	321 (19.1%)	
Yes, 5–10 cigarettes per day	105 (6.2%)	54 (3.2%)	
Yes, more than 10 cigarettes per day	145 (8.6%)	119 (7.1%)	
**Sleep**			**<0.001** *
<7 h per night	733 (43.5%)	415 (24.6%)	
7–9 h per night	850 (50.5%)	915 (54.4%)	
>9 h per night	101 (6.0%)	354 (21.0%)	

*Marginal homogeneity test; numbers in bold indicate significant p-values.

### Bivariate analysis

Older age was significantly but weakly, associated with a higher MEDAS score (r = 0.05; p = 0.035), however, the MEDAS score was not significantly associated with any of the other sociodemographic characteristics collected (Table 5).

**Table 5:** Bivariate analysis of factors associated with the MEDAS score.

Variable	MEDAS score (mean ± SD)	*p*
**Gender**		0.742*
Male	7.45 ± 2.41	
Female	7.41 ± 2.19	
**Marital status**		0.547*
Single / divorced / widowed	7.41 ± 2.27	
Married	7.50 ± 2.21	
**District**		0.182**
Beirut	7.25 ± 2.29	
Mount Lebanon	7.30 ± 2.15	
North	7.55 ± 2.35	
South	7.44 ± 2.29	
Bekaa	7.61 ± 2.21	
**Body Mass Index categories**		0.821**
Underweight	7.55 ± 2.50	
Normal	7.43 ± 2.17	
Overweight	7.41 ± 2.42	
Obese	7.28 ± 2.21	
**Body Mass Index (continuous)**	r = -0.02	0.495***
**Age**	r = 0.05	**0.035** ***

*Student t test

**ANOVA test

***Pearson test; numbers in bold indicate significant *p* values; r = Pearson correlation coefficient.

### Multivariable analysis

A stepwise linear regression, taking the continuous MEDAS score as the dependent variable, was conducted; variables entered in the model were age and district of residency. The results showed that older age (Beta = 0.02; p = 0.035; 95% CI 0.001–0.03) was significantly associated with a higher MEDAS score.

### Gender differences

Mean body mass index (BMI) score was significantly higher in males when compared to females (25.89 ± 5.42 vs 22.60 ± 3.82; p<0.001), but no difference was found between genders in terms of the MEDAS score (7.45 ± 2.41 vs 7.41 ± 2.19; p = 0.742).

## Discussion

The current study provides valuable insight about dietary intake, MD adherence and lifestyle habits during the lockdown that was imposed because of the COVID-19 pandemic in Lebanon. Significant changes in sleep quantity and dietary intake were reported. The only variable that was found to be associated with MD adherence was older age.

More than 40% of our study sample reported that their dietary intake had changed negatively because of the pandemic and its restrictions, whereas almost a quarter of the participants described positive improvements in their diets. Our findings are in line with several other studies that have been conducted in different parts of the world. For example, Di Renzo and colleagues reported that amongst a sample of Italians, 37.2% felt that they were following inferior dietary intakes and 16.7% felt that they had improved their dietary habits, due to lockdowns that had taken place because of COVID-19 [15]. Additionally, in a study that took place in Kuwait, Husain & Ashkanani indicated significant changes in eating practices among their Kuwaiti sample [29]. Furthermore, Radwan et al. also reported that 31.8% of Emiratis increased food consumption [30]. Cheikh et al also reported that close to 40% of the Lebanese participants in their study were not consuming fruits and vegetables on a daily basis [31], while Dimassi et al reported a significant increase in consumption of fruits and vegetables [32]. Nevertheless, as a result of the pandemic, almost 40% of the participants have reported weight gain almost [33]. Unhealthy dietary patterns and weight gain are especially problematic during a pandemic since healthy dietary habits are the cornerstone to enhanced immunity, which could help ward of the severe consequences of viruses [34].

We also found that participants in this study reported smoking less during the pandemic. This is similar to the results reported by Di Renzo and colleagues who also described that smoking frequency was reduced in their study sample during the lockdown [15]. This is a positive outcome that may be attributed to the smokers’ concern about COVID-19-related respiratory distress, which led to lower exposure to smoking [35].

Additionally, we found that participants reported sleeping more during the pandemic. This could be due to lockdowns and restrictions that were imposed and changes that resulted in virtual workplaces. Getting adequate sleep may be a positive finding since improved sleep improves immunity [36]. It may also be a negative finding since increased sleep hours are associated with psychiatric disease conditions and a higher BMI [37]. Unfortunately, we did not assess sleep quality and our findings are based simply on the number of hours of sleep reported during the pandemic. Likewise, Di Renzo and colleagues also reported increased sleep hours during lockdown among the study participants [15]. Future studies should focus on assessing the quality of sleep during a lockdown and its effect on dietary intake and immunity.

In terms of the MD dietary pattern, only a small number of participants reported high adherence and only age was positively correlated with a higher MEDAS score, which is a common finding in the scientific literature [17,38–40]. However, increased adherence to the MD diet was not inversely correlated with BMI which was a surprising finding since many studies have reported contradicting results [38,41,42]. We hypothesize that this could be a result of increased food intake among our sample, secondary to COVID-19 restrictions, regardless of the quality of the diet as it was previously noted that lockdowns are associated with increased preparation of homemade meals more frequently [43–45].

Consuming more portions of fruits, legumes, olive oil, nuts and wine would give a higher MEDAS score, which suggests more adherence but could also result in increased weight because of increased caloric intake. This was an interesting finding in our study, as over a third of the individuals reported increasing their intake of fruits, vegetables, and grains. Weight gain is more likely to occur when food intake is increased while physical activity levels decline, as is often the case during lockdowns [33,43]. Although we did not assess physical activity using validated tools, similar studies have found that physical activity levels often fall during lockdown [43]. Future studies should investigate this association further [16,46].

### Implications

Hence, it is easy to conclude that the lockdowns and closures imposed by governments worldwide have had some impact on the nutritional behavior in a significant number of people and the picture is no different in Lebanon. Since nutritional interventions may have a role in COVID-19 infection and mortality rates, our results should be used to guide public health policymakers to provide programs that focus on sending appropriate nutritional advice to better help in the management and prevention of the pandemic consequences [17].

### Limitations and strengths

Our study is not without limitations, as our design was cross-sectional, which does not allow us to confer causation. Participants recruited had to self-report and describe lifestyle habits, and anthropometric parameters as our survey was web-based and thus respondents may not describe their dietary intake adequately. Additionally, the questionnaire was sent out in English, and although this could lead to a sampling bias, it is worth noting that a great proportion of the Lebanese population is fluent in the English language. Besides, the Cronbach’s alpha value was low, therefore, results should be interpreted with caution. Furthermore, our sample had a high number of female respondents compared to males. Yet, research has consistently documented that females are more likely to participate in surveys [47]. Our results should be interpreted with caution since correlations were significant, but weak.

However, our study has several strengths that are worthy to mention. First, our study was adequately powered, as we were able to recruit a large number of participants. Additionally, the questionnaire that was used was validated, thorough, and detailed. Furthermore, we were able to recruit participants from all different governorates in Lebanon, which makes the study somewhat representative of the Lebanese population. To our knowledge, this is also the first study of its kind to be conducted in Lebanon that examines how lockdowns and countrywide closures affect people’s lifestyle changes and adherence to the MD.

## Conclusion

This study assessed lifestyle, dietary habits and MD adherence among a Lebanese sample during lockdown. We concluded from the findings in this study that there is room for improvement in the Lebanese dietary and lifestyle habits as MD adherence was suboptimal during the COVID-19 lockdown. It is critical that the Lebanese government implements public health programs in order to promote awareness about the importance of adhering to a healthy lifestyle and making appropriate dietary and lifestyle choices.

## References

[1] Wang C.; Horby P.W.; Hayden F.G.; Gao G.F. A novel coronavirus outbreak of global health concern. *Lancet (London, England)* 2020, 395, 470–473, doi: 10.1016/S0140-6736(20)30185-9 31986257 PMC7135038

[2] Celorio-Sardà R.; Comas-Basté O.; Latorre-Moratalla M.L.; Zerón-Rugerio M.F.; Urpi-Sarda M.; Illán-Villanueva M.; et al. Effect of COVID-19 Lockdown on Dietary Habits and Lifestyle of Food Science Students and Professionals from Spain. *Nutrients* 2021, 13, doi: 10.3390/nu13051494 33924965 PMC8146598

[3] Izzo L.; Santonastaso A.; Cotticelli G.; Federico A.; Pacifico S.; Castaldo L.; et al. An Italian Survey on Dietary Habits and Changes during the COVID-19 Lockdown. *Nutrients* 2021, 13, doi: 10.3390/nu13041197 33916384 PMC8065756

[4] Sepúlveda-Loyola W.; Rodríguez-Sánchez I.; Pérez-Rodríguez P.; Ganz F.; Torralba R.; Oliveira D.V.; et al. Impact of Social Isolation Due to COVID-19 on Health in Older People: Mental and Physical Effects and Recommendations. *The journal of nutrition, health & aging* 2020, 24, 938–947, doi: 10.1007/s12603-020-1469-2 33155618 PMC7597423

[5] Perszyk E.E.; Hutelin Z.; Trinh J.; Kanyamibwa A.; Fromm S.; Davis X.S.; et al. Fat and carbohydrate interact to potentiate food reward in healthy weight but not in overweight or obesity. *bioRxiv* 2021, 2021.2001.2029.428845, doi: 10.1101/2021.01.29.428845 PMC8067354 33917347

[6] Torres S.J.; Nowson C.A. Relationship between stress, eating behavior, and obesity. *Nutrition (Burbank, Los Angeles County, Calif.)* 2007, 23, 887–894, doi: 10.1016/j.nut.2007.08.008 17869482

[7] Ma Y.; Ratnasabapathy R.; Gardiner J. Carbohydrate craving: not everything is sweet. *Current opinion in clinical nutrition and metabolic care* 2017, 20, 261–265, doi: 10.1097/MCO.0000000000000374 28375878 PMC5837018

[8] Füzéki E.; Groneberg D.A.; Banzer W. Physical activity during COVID-19 induced lockdown: recommendations. *Journal of occupational medicine and toxicology (London, England)* 2020, 15, 25, doi: 10.1186/s12995-020-00278-9 32817753 PMC7422663

[9] Schultchen D.; Reichenberger J.; Mittl T.; Weh T.R.M.; Smyth J.M.; Blechert J.; et al. Bidirectional relationship of stress and affect with physical activity and healthy eating. *British journal of health psychology* 2019, 24, 315–333, doi: 10.1111/bjhp.12355 30672069 PMC6767465

[10] Kharroubi S.; Saleh F. Are Lockdown Measures Effective Against COVID-19? *Frontiers in public health* 2020, 8, 549692, doi: 10.3389/fpubh.2020.549692 33194950 PMC7642264

[11] Nasreddine L.; Naja F.A.; Sibai A.M.; Helou K.; Adra N.; Hwalla N. Trends in nutritional intakes and nutrition-related cardiovascular disease risk factors in Lebanon: the need for immediate action. Le Journal medical libanais. *The Lebanese medical journal* 2014, 62, 83–91, doi: 10.12816/0004102 25011369

[12] Di Renzo L.; Gualtieri P.; Romano L.; Marrone G.; Noce A.; Pujia A.; et al. Role of Personalized Nutrition in Chronic-Degenerative Diseases. *Nutrients* 2019, 11, doi: 10.3390/nu11081707 31344895 PMC6723746

[13] De Lorenzo A.; Bernardini S.; Gualtieri P.; Cabibbo A.; Perrone M.A.; Giambini I.; et al. Mediterranean meal versus Western meal effects on postprandial ox-LDL, oxidative and inflammatory gene expression in healthy subjects: a randomized controlled trial for nutrigenomic approach in cardiometabolic risk. *Acta diabetologica* 2017, 54, 141–149, doi: 10.1007/s00592-016-0917-2 27709360

[14] Widmer R.J.; Flammer A.J.; Lerman L.O.; Lerman A. The Mediterranean diet, its components, and cardiovascular disease. *The American journal of medicine* 2015, 128, 229–238, doi: 10.1016/j.amjmed.2014.10.014 25447615 PMC4339461

[15] Di Renzo L.; Gualtieri P.; Pivari F.; Soldati L.; Attinà A.; Cinelli G.; et al. Eating habits and lifestyle changes during COVID-19 lockdown: an Italian survey. *Journal of translational medicine* 2020, 18, 229, doi: 10.1186/s12967-020-02399-5 32513197 PMC7278251

[16] Kolokotroni O.; Mosquera M.C.; Quattrocchi A.; Heraclides A.; Demetriou C.; Philippou E. Lifestyle habits of adults during the COVID-19 pandemic lockdown in Cyprus: evidence from a cross-sectional study. *BMC Public Health* 2021, 2i1, 786, doi: 10.1186/s12889-021-10863-0 33892688 PMC8064698

[17] Rodriguez-Leyva D.; Pierce G.N. The Impact of Nutrition on the COVID-19 Pandemic and the Impact of the COVID-19 Pandemic on Nutrition. *Nutrients* 2021, 13, 1752. doi: 10.3390/nu13061752 34064053 PMC8223988

[18] Della Valle P.G.; Mosconi G.; Nucci D.; Vigezzi G.P.; Gentile L.; Gianfredi V.; et al. Adherence to the Mediterranean Diet during the COVID-19 national lockdowns: A systematic review of observational studies. *Acta Bio Medica: Atenei Parmensis* 2021, 92. 10.23750/abm.v92iS6.12233 PMC8851000 34739464

[19] Papadaki A.; Johnson L.; Toumpakari Z.; England C.; Rai M.; Toms S.; et al. Validation of the English Version of the 14-Item Mediterranean Diet Adherence Screener of the PREDIMED Study, in People at High Cardiovascular Risk in the UK. *Nutrients* 2018, 10, doi: 10.3390/nu10020138 29382082 PMC5852714

[20] Garcia-Conesa M.T.; Philippou E.; Pafilas C.; Massaro M.; Quarta S.; Andrade V.; et al. Exploring the Validity of the 14-Item Mediterranean Diet Adherence Screener (MEDAS): A Cross-National Study in Seven European Countries around the Mediterranean Region. *Nutrients* 2020, 12, doi: 10.3390/nu12102960 32992649 PMC7601687

[21] Hebestreit K.; Yahiaoui-Doktor M.; Engel C.; Vetter W.; Siniatchkin M.; Erickson N.; et al. Validation of the German version of the Mediterranean Diet Adherence Screener (MEDAS) questionnaire. *BMC Cancer* 2017, 17, 341, doi: 10.1186/s12885-017-3337-y 28521737 PMC5437541

[22] Mahdavi-Roshan M.; Salari A.; Soltanipour S. Reliability and Validity of the 14-point mediterranean diet adherence screener among the Iranian high risk population. *Mediterranean Journal of Nutrition and Metabolism* 2018, 11, 323–329.

[23] Fontalba-Romero M.I.; Lopez-Enriquez S.; Lago-Sampedro A.; Garcia-Escobar E.; Pastori R.L.; Dominguez-Bendala J.; et al. Association between the Mediterranean Diet and Metabolic Syndrome with Serum Levels of miRNA in Morbid Obesity. *Nutrients* 2021, 13, doi: 10.3390/nu13020436 33572759 PMC7911421

[24] Schröder H.; Fitó M.; Estruch R.; Martínez‐González M.A.; Corella D.; Salas‐Salvadó J.; et al. A short screener is valid for assessing Mediterranean diet adherence among older Spanish men and women. *The Journal of nutrition* 2011, 141, 1140–1145. doi: 10.3945/jn.110.135566 21508208

[25] Czarniecka-Skubina E.; Pielak M.; Sałek P.; Głuchowski A.; Kobus-Cisowska J.; Owczarek T. Use of Food Services by Consumers in the SARS-CoV-2 Pandemic. *How the Eating Habits of Consumers Changed in View of the New Disease Risk Factors? Nutrients* 2021, 13, 2760. 34444920 10.3390/nu13082760 PMC8400554

[26] Mattavelli E.; Olmastroni E.; Bonofiglio D.; Catapano A.L.; Baragetti A.; Magni P. Adherence to the Mediterranean diet: impact of geographical location of the observations. *Nutrients* 2022, 14, 2040. doi: 10.3390/nu14102040 35631181 PMC9144454

[27] Hair J.F. Jr; Hult G.T.M.; Ringle C.M.; Sarstedt M. *A primer on partial least squares structural equation modeling (PLS-SEM)*; Sage publications: 2021.

[28] Bursac Z.; Gauss C.H.; Williams D.K.; Hosmer D.W. Purposeful selection of variables in logistic regression. *Source Code Biol Med* 2008, 3, 17, doi: 10.1186/1751-0473-3-17 19087314 PMC2633005

[29] Husain W.; Ashkanani F. Does COVID-19 change dietary habits and lifestyle behaviours in Kuwait: a community-based cross-sectional study. *Environmental health and preventive medicine* 2020, 25, 61, doi: 10.1186/s12199-020-00901-5 33045996 PMC7548533

[30] Radwan H.; Al Kitbi M.; Hasan H.; Al Hilali M.; Abbas N.; Hamadeh R.; et al. Indirect Health Effects of COVID-19: Unhealthy Lifestyle Behaviors during the Lockdown in the United Arab Emirates. *International journal of environmental research and public health* 2021, 18, doi: 10.3390/ijerph18041964 33670510 PMC7922937

[31] Cheikh Ismail L.; Hashim M.; Mohamad M.N.; Hassan H.; Ajab A.; Stojanovska L.; et al. Dietary Habits and Lifestyle During Coronavirus Pandemic Lockdown: Experience From Lebanon. *Front Nutr* 2021, 8, 730425, doi: 10.3389/fnut.2021.730425 34527692 PMC8435593

[32] Dimassi H.; Haddad R.; Awada R.; Mattar L.; Hassan H.F. Food shopping and food hygiene related knowledge and practices during the COVID-19 pandemic: The case of a developing country. *Italian Journal of Food Safety* 2021, 10. doi: 10.4081/ijfs.2021.9384 34497780 PMC8404529

[33] Cheikh Ismail L.; Osaili T.M.; Mohamad M.N.; Al Marzouqi A.; Habib-Mourad C.; Abu Jamous D.O.; et al. Assessment of Dietary and Lifestyle Responses After COVID-19 Vaccine Availability in Selected Arab Countries. *Front Nutr* 2022, 9, 849314, doi: 10.3389/fnut.2022.849314 35495916 PMC9048021

[34] Butler M.J.; Barrientos R.M. The impact of nutrition on COVID-19 susceptibility and long-term consequences. *Brain, behavior, and immunity* 2020, 87, 53–54, doi: 10.1016/j.bbi.2020.04.040 32311498 PMC7165103

[35] Brake S.J.; Barnsley K.; Lu W.; McAlinden K.D.; Eapen M.S.; Sohal S.S. Smoking Upregulates Angiotensin-Converting Enzyme-2 Receptor: A Potential Adhesion Site for Novel Coronavirus SARS-CoV-2 (Covid-19). *Journal of clinical medicine* 2020, 9, doi: 10.3390/jcm9030841 32244852 PMC7141517

[36] Silva E.; Ono B.; Souza J.C. Sleep and immunity in times of COVID-19. *Rev Assoc Med Bras (1992)* 2020, 66 Suppl 2, 143–147, doi: 10.1590/1806-9282.66.S2.143 32965373

[37] Léger D.; Beck F.; Richard J.-B.; Sauvet F.; Faraut B. The risks of sleeping “too much”. Survey of a national representative sample of 24671 adults (INPES health barometer). *PloS one* 2014, 9, e106950. doi: 10.1371/journal.pone.0106950 25226585 PMC4165901

[38] Jurado D.; Burgos-Garrido E.; Diaz F.J.; Martínez-Ortega J.M.; Gurpegui M. Adherence to the Mediterranean dietary pattern and personality in patients attending a primary health center. *Journal of the Academy of Nutrition and Dietetics* 2012, 112, 887–891, doi: 10.1016/j.jand.2012.02.026 22709815

[39] Bagues A.; Almagro A.; Bermúdez T.; López-Tofiño Y.; González A.; Abalo R. Adherence to the Mediterranean diet: An online questionnaire based-study in a Spanish population sample just before the Covid-19 lockdown. *Functional Foods in Health and Disease* 2021, 11, 283–294.

[40] Giacalone D.; Frost M.B.; Rodriguez-Perez C. Reported Changes in Dietary Habits During the COVID-19 Lockdown in the Danish Population: The Danish COVIDiet Study. *Front Nutr* 2020, 7, 592112, doi: 10.3389/fnut.2020.592112 33364250 PMC7752855

[41] Martin-Calvo N.; Chavarro J.E.; Falbe J.; Hu F.B.; Field A.E. Adherence to the Mediterranean dietary pattern and BMI change among US adolescents. *International Journal of Obesity* 2016, 40, 1103–1108, doi: 10.1038/ijo.2016.59 27102053 PMC4935550

[42] Martínez-González M.A.; García-Arellano A.; Toledo E.; Salas-Salvadó J.; Buil-Cosiales P.; Corella D.; et al. A 14-item Mediterranean diet assessment tool and obesity indexes among high-risk subjects: the PREDIMED trial. *PloS one* 2012, 7, e43134, doi: 10.1371/journal.pone.0043134 22905215 PMC3419206

[43] Boleslawska I.; Blaszczyk-Bebenek E.; Jagielski P.; Jagielska A.; Przyslawski J. Nutritional behaviors of women and men in Poland during confinement related to the SARS-CoV-2 epidemic. *Sci Rep* 2021, 11, 19984, doi: 10.1038/s41598-021-99561-w 34620981 PMC8497511

[44] Sanchez-Sanchez E.; Ramirez-Vargas G.; Avellaneda-Lopez Y.; Orellana-Pecino J.I.; Garcia-Marin E.; Diaz-Jimenez J. Eating Habits and Physical Activity of the Spanish Population during the COVID-19 Pandemic Period. *Nutrients* 2020, 12, doi: 10.3390/nu12092826 32942695 PMC7551353

[45] Tribst A.A.L.; Tramontt C.R.; Baraldi L.G. Factors associated with diet changes during the COVID-19 pandemic period in Brazilian adults: Time, skills, habits, feelings and beliefs. *Appetite* 2021, 163, 105220. doi: 10.1016/j.appet.2021.105220 33785430

[46] Dor-Haim H.; Katzburg S.; Revach P.; Levine H.; Barak S. The impact of COVID-19 lockdown on physical activity and weight gain among active adult population in Israel: a cross-sectional study. *BMC Public Health* 2021, 21, 1521, doi: 10.1186/s12889-021-11523-z 34362319 PMC8343341

[47] Curtin R.; Presser S.; Singer E. The effects of response rate changes on the index of consumer sentiment. *Public opinion quarterly* 2000, 64, 413–428, doi: 10.1086/318638 11171024

